# Differential Modulation of Capacitation-Associated Responses in Boar Spermatozoa by In Vitro Capacitation Media: A Kinetic Study

**DOI:** 10.3390/ani16162553

**Published:** 2026-08-15

**Authors:** Eduardo de Mercado, Irene Ortega, Adrián Martín-San Juan, Helena Nieto-Cristóbal, María José Martinez-Alborcia, Miguel Ángel Silvestre, Manuel Álvarez-Rodríguez

**Affiliations:** 1 Department of Animal Reproduction, Spanish National Institute for Agricultural and Food Research and Technology (INIA-CSIC), 28040 Madrid, Spainadrian.martin@inia.csic.es (A.M.-S.J.); helena.nieto@inia.csic.es (H.N.-C.); manuel.alvarez@inia.csic.es (M.Á.-R.); 2 Topigs Norsvin España SLU-AIM Ibérica, 28290 Las Rozas, Spain; mariajose.alborcia@aimiberica.com; 3 Department of Cellular Biology, Functional Biology and Physical Anthropology, University of Valencia, 46110 Burjassot, Spain; miguel.silvestre@uv.es

**Keywords:** sperm, porcine, capacitation, medium, sperm quality

## Abstract

In vitro capacitation of boar spermatozoa remains highly variable among laboratories due to differences in media composition, incubation conditions, and previous semen handling. Since capacitation is a dynamic process, identifying how different media modulate specific sperm responses over time is crucial to improving the standardization of porcine in vitro fertilization protocols. This study evaluated the temporal dynamics of membrane lipid disorder, acrosomal integrity, mitochondrial activity, and motility patterns in response to different media. Commercial boar semen doses were centrifuged and resuspended in a non-capacitating control solution or in three capacitation media differing mainly in bicarbonate, caffeine, and energy substrate composition, and evaluated every 15 min using flow cytometry and computer-assisted semen analysis at 38 °C for up to 2 h. Media containing higher bicarbonate and caffeine concentrations induced rapid membrane lipid destabilization and increased acrosomal exocytosis during the initial incubation period. Subsequently, these changes were accompanied by a more rapid decline in cell viability over time. In contrast, the medium containing lower bicarbonate together with lactate and pyruvate preserved higher viability, mitochondrial activity, and a more sustained, linear movement pattern (higher linearity and straightness). Interestingly, training-associated responses were detected immediately at time 0, suggesting that semen storage conditions may prime spermatozoa before incubation. Overall, medium composition and pre-treatment handling conditions should both be considered when standardizing porcine reproductive technologies.

## Simple Summary

Designing successful systems for artificial breeding in swine depends heavily on understanding how sperm cells prepare to fertilize an egg. However, this activation process is far from being standardized. Laboratories worldwide use different incubation liquids and handling procedures, leading to inconsistent outcomes. In this study, we compared how boar sperm respond over time when exposed to three common capacitation media. Our results revealed that certain formulations trigger a very rapid response, but also shorten sperm lifespan. Conversely, another medium maintains sperm movement and quality for longer periods, showing a more gradual response. Interestingly, some cell changes were detectable right after processing, even in a control solution, suggesting that previous storage conditions already alter the spermatozoa before incubation starts. Our findings highlight that both incubation media and previous semen management can influence sperm capacitation. A better understanding of these combined factors will help standardize laboratory procedures and improve reproductive technologies in pigs.

## 1. Introduction

For mammalian spermatozoa to acquire fertilizing ability, they must undergo a series of biochemical and functional modifications collectively known as capacitation [1,2]. Capacitation is not a single event but a dynamic and progressive process involving membrane lipid remodeling, cholesterol efflux, ion fluxes, intracellular signaling activation and changes in sperm motility patterns that ultimately enable acrosomal exocytosis and oocyte fertilization [3,4]. While these physiological changes are naturally coordinated within the female reproductive tract, replicating and modulating them consistently under in vitro conditions represents a critical hurdle in swine reproduction.

Furthermore, in boar sperm, capacitation is particularly complex due to the high sensitivity of their sperm membranes to environmental conditions and the high susceptibility of this species to membrane destabilization and spontaneous acrosomal exocytosis [5]. In commercial swine production, the artificial conditions associated with liquid semen storage at 15–17 °C may promote premature capacitation-associated changes. This early, sublethal destabilization of the sperm plasma membrane compromises sperm longevity and alters the basal responsiveness of spermatozoa to subsequent stimuli, even before the onset of any experimental incubation [5].

To replicate and control this process in vitro, defined culture media are used, but their standardization remains a major challenge due to the wide variety of incubation conditions, media compositions, and co-incubation times used by different laboratories for in vitro fertilization [6,7,8,9]. These differences are particularly relevant in pigs, where excessive or prolonged capacitation conditions can promote premature acrosomal exocytosis, reduced sperm viability, and increased polyspermy rates during IVF procedures [7,9,10]. Therefore, understanding how to achieve a balance between efficient sperm capacitation and the maintenance of sperm functionality over time remains one of the main challenges in porcine reproductive biotechnology.

Many authors agree that there are four essential components for sperm capacitation: bicarbonate, calcium, and a cholesterol acceptor such as bovine serum albumin (BSA) [11,12]; the fourth—caffeine—although not physiologically present in the genital tract, has been used as a capacitation agent due to its effect on intracellular signaling [13]. Among these factors, bicarbonate concentration and the nature of the available energy substrates are thought to play a major role in determining the kinetics of the capacitation process. Higher bicarbonate concentrations generally promote faster capacitation-associated membrane remodeling, although often at the expense of sperm viability. By contrast, lower concentrations (around 15 mM) have been associated with efficient capacitation while reducing polyspermy during in vitro fertilization [11,14]. Similarly, while glucose can limit the full acquisition of the capacitated state, oxidative substrates like pyruvate and lactate appear far more favorable for maintaining prolonged sperm functionality [15].

The diversity of formulations used, incubation times, and experimental conditions has made it difficult to standardize an effective in vitro protocol for porcine sperm capacitation. For this reason, in this study, we compared three widely used capacitation media that share common base components but differ notably in the presence or concentration of bicarbonate, caffeine, and energy substrates.

CAP medium, derived from the medium of Brackett and Oliphant (1975) [16] adapted for swine [14,15,17] and recently applied in our laboratory in other experimental studies [7], contains high concentrations of bicarbonate, caffeine, and glucose. TALP medium, a modified version of Tyrode’s medium [6,18,19,20], includes lower concentrations of bicarbonate, caffeine and glucose. Finally, TYR medium, a classical Tyrode’s formulation [8,21,22,23], contains the lowest bicarbonate concentration, lacks caffeine and uses sodium lactate as its main energy substrate.

Therefore, the aim of this study was to characterize the temporal dynamics of several capacitation-associated responses in boar spermatozoa incubated for 2 h at 38 °C in three widely used capacitation media (CAP, TALP and TYR). To achieve this, sperm membrane lipid disorder and integrity, acrosomal status, mitochondrial activity and sperm motility were evaluated at multiple time points using flow cytometry and CASA.

## 2. Materials and Methods

### 2.1. Ethics Statement

Commercial boar semen doses were sourced from the artificial insemination (AI) center AIM Ibérica (Topigs Norsvin Spain SLU; León, Spain). All sample logistics adhered strictly to relevant European (Directive 2010/63/EU; ES13RS04P, July 2012) and Spanish (ES300130640127, August 2006) regulatory frameworks for the processing and distribution of AI doses, guaranteeing compliance with established biosecurity and animal welfare protocols. Because this investigation relied exclusively on commercial doses harvested from production boars under routine husbandry, no direct animal handling or invasive experimental procedures were conducted. Consequently, formal institutional ethical approval was exempt in accordance with Spanish legislation (Real Decreto 53/2013) regarding standard zootechnical practices.

### 2.2. Reagents and Media

Unless otherwise specified, all chemicals and reagents were obtained from Sigma-Aldrich, St. Louis, MO, USA. For this study, three different sperm capacitation extenders were used: CAP, TALP, and TYR extender. The specific composition of each extender is detailed in Table 1. In addition, phosphate-buffered saline (PBS; cat. no. P4417, Sigma-Aldrich, St. Louis, MO, USA) was used as a non-capacitating control medium (pH 7.4; 285 mOsm/kg).

### 2.3. Animal Handling and Sperm Collection

For this study, semen samples from sixteen fertile Swedish Landrace boars with proven fertility, maintained under controlled environmental conditions and receiving an appropriate commercial diet tailored for breeding boars were used. The ejaculates were collected using the double-glove method and immediately diluted in a commercial long-term extender (Duragen; MAGAPOR S.L., Zaragoza, Spain) to a final concentration of 2.4 × 10^9^ spermatozoa per 80 mL dose and stored at a controlled temperature between 15 and 17 °C for 24–36 h before experimental use. To minimize individual male variation and eliminate potential “boar effects” that could bias sperm responses to artificial stimuli, samples were grouped into eight pools, each composed of semen from two different boars. The boars selected for this study were previously confirmed to exhibit optimal semen quality, defined as a minimum of 80% total motility and under 15% morphologically abnormal spermatozoa, as well as proven fertility.

### 2.4. Experimental Design

A 10 mL aliquot from each pool was centrifuged at 1500× *g* for 5 min at 17 °C to remove seminal plasma and the refrigeration extender. The resulting sperm pellets were resuspended in an equal volume of one of the three capacitation media (CAP, TALP, and TYR), as well as in a fourth treatment consisting of phosphate-buffered saline (PBS, cat. no. P4417, Sigma-Aldrich, St. Louis, MO, USA), which served as a non-capacitating control. PBS was selected to provide an isotonic, pH-stable, and unsupplemented saline baseline that avoids inducing active capacitation cascades or metabolic shifts [24].

After resuspension, samples were incubated under capacitating conditions at 38 °C for 2 h in a single sealed 15 mL polypropylene tube per treatment, with minimal headspace to maintain media stability and prevent evaporation, ensuring stable pH conditions throughout the incubation period. To counteract sperm sedimentation during elongated incubation, each tube was gently inverted and homogenized immediately before every sample aliquot extraction. Basal sperm quality and kinetic parameters were first evaluated before centrifugation or treatment exposure directly from the preserved commercial doses, establishing the pre-capacitation state (Prev). Following resuspension in the experimental media, sperm quality parameters were evaluated by flow cytometry at time 0 (immediately after resuspension) and every 15 min thereafter until the end of incubation. Sperm motility was assessed at 0, 1, and 2 h using a Computer-Assisted Semen Analysis (CASA).

### 2.5. Sperm Analyses

#### 2.5.1. Flow Cytometry Analysis

Flow cytometry assessments were performed on a CytoFLEX platform (Beckman Coulter, Brea, CA, USA) operating with 405 nm (violet), 488 nm (blue), and 640 nm (red) lasers. Signal acquisition was captured through 450/45 BP, 525/40 BP, 585/42 BP, 610/20 BP, and 660/20 BP emission filters. Fluorescence signals were collected using logarithmic amplification, while FSC and SSC were displayed in linear mode. Instrument performance and optical alignment were verified daily with calibration beads (CytExpert, Beckman Coulter). The spermatozoa population was initially gated on forward (FSC) versus side scatter (SSC) profiles to filter out non-sperm debris, followed by doublet exclusion using FSC-area (FSC-A) against FSC-height (FSC-H) plot gating. The main sperm population included >95% of singlet events. Samples were analyzed at a sheath flow rate of 10 µL/min, with a final sperm concentration of 1–2 × 10^6^ cells/mL to minimize coincident events. A maximum acquisition speed of 400–800 events/s was used, and data from 10,000 spermatozoa per sample were collected. Hoechst 33342 (cat. no. H1399, Molecular Probes™, Invitrogen, Thermo Fisher Scientific, Eugene, OR, USA. Working solution: 0.41 µM) was also included in all analyses to identify the nucleus of spermatozoa and exclude debris from the analyses, and fluorescence was detected in the ultraviolet channel (450/45 BP). Fluorescence compensation was applied using single-stained controls for each fluorochrome. All data were processed using Kaluza Analysis Software v2.1 (Beckman Coulter).

##### Viability and Acrosome Integrity

Sperm viability and acrosomal integrity were evaluated by flow cytometry using YoPro-1 (Yop; cat. no. Y3603, Thermo Fisher Scientific, Waltham, MA, USA. Working solution: 0.4 µM) and fluorescein isothiocyanate-conjugated peanut agglutinin Alexa Fluor™ 568 Conjugate (PNA; cat. no. L32458, Thermo Fisher Scientific, Waltham, MA, USA. Working solution: 0.009 µM) (Ref. [25]; with modifications for Alexa Fluor™ 568). YoPro-1 fluorescence was detected in the green channel (525 nm) and PNA-FITC in the orange/red channel (585 nm). Sperm were incubated with the fluorochromes for 10 min at 37 °C in the dark. Sperm were classified into four subpopulations: viable sperm with intact acrosome (Yop–/PNA–), viable sperm with reacted acrosome (Yop–/PNA+), non-viable sperm with intact acrosome (Yop+/PNA–), and non-viable sperm with reacted acrosome (Yop+/PNA+). Results were expressed as the percentage of each subpopulation within the total gated sperm population.

##### Viability and Membrane Lipid Disorder

Sperm viability and plasma membrane lipid organization were assessed by flow cytometry using YoPro-1 (Yop; cat. no. Y3603. Thermo Fisher Scientific, Waltham, MA, USA. Working solution: 0.4 µM) and Merocyanine 540 (M540; cat. no. M24571, Thermo Fisher Scientific, Waltham, MA, USA. Working solution: 0.49 µM) [25]. YoPro-1 fluorescence was detected in the green channel (525 nm) and M540 in the orange channel (585 nm). Sperm were incubated with the fluorochromes for 10 min at 37 °C in the dark. Sperm were classified into four subpopulations: viable sperm with a stable plasma membrane (Yop–/M540–), viable sperm with an unstable plasma membrane (Yop–/M540+), non-viable sperm with a stable plasma membrane (Yop+/M540–), and non-viable sperm with an unstable plasma membrane (Yop+/M540+). Results were expressed as the percentage of each subpopulation within the total gated sperm population.

##### Viability and Mitochondrial Activity

Sperm viability and mitochondrial activity were evaluated by flow cytometry using YoPro-1 (Yop; cat. no. Y3603. Thermo Fisher Scientific, Waltham, MA, USA. Working solution: 0.4 µM) and Mitotracker Deep Red (MTdr; cat. no. M22426 Thermo Fisher Scientific, Waltham, MA, USA. Working solution: 0.0625 µM) [25]. YoPro-1 fluorescence was detected in the green channel (525 nm) and MTdr in the far-red channel (660 nm). Sperm were incubated with the fluorochromes for 10 min at 37 °C in the dark. Sperm were classified into four subpopulations: viable sperm without mitochondrial activity (Yop–/MTdr–), viable sperm with mitochondrial activity (Yop–/MTdr+), non-viable sperm without mitochondrial activity (Yop+/MTdr–), and non-viable sperm with mitochondrial activity (Yop+/MTdr+). Results were expressed as the percentage of each subpopulation within the total gated sperm population.

#### 2.5.2. Sperm Motility

For sperm motility analysis, 5 μL aliquots of each sample were loaded onto a Makler chamber pre-warmed to 37 °C and examined using a Computer-Assisted Semen Analysis (CASA) system (AI Station 1.2.24, SPERMTECH^®^, Buñol, Spain) coupled to a Nikon Eclipse Si RS phase contrast microscope at 100× magnification. A minimum of five non-overlapping video fields were recorded per sample, with at least 200 spermatozoa analyzed in total. Each field comprised 50 captured frames. Sperm detection criteria were set with a minimum cell area of 10 μm^2^ and a maximum of 69 μm^2^, while progressive motility was defined by a straightness threshold of 50%. The parameters assessed included total motility (TM, %), progressive motility (PM, %), curvilinear velocity (VCL, µm/s), straight-line velocity (VSL, µm/s), average path velocity (VAP, µm/s), linearity (LIN, %), straightness (STR, %), and wobble coefficient (WOB, %).

### 2.6. Statistical Analysis

Statistical processing was executed in GraphPad Prism (v. 8.4.3; GraphPad Software, San Diego, CA, USA). Data compliance with parametric model assumptions was confirmed prior to hypothesis testing: normality was evaluated via the Shapiro–Wilk test, and homoscedasticity of variance was verified using Levene’s test. As all variables met parametric assumptions, data were analyzed using two-way repeated-measures ANOVA to evaluate the main effects of treatment, incubation time, and their potential interactions, considering time as the repeated factor. The ‘Prev’ value was included in the analysis as a baseline reference of the sperm physiological status prior to capacitation incubation. Tukey’s post hoc test was subsequently applied for multiple-comparison analysis to determine significant differences among treatments at each specific time point, as well as to evaluate the effect of incubation time within each individual group. Each heterospermic pool (*n* = 8) was considered the experimental unit for all statistical evaluations. Differences were considered statistically significant when *p* < 0.05. Data are presented as means ± standard errors of the mean (SEM).

## 3. Results

**Figure 1:**
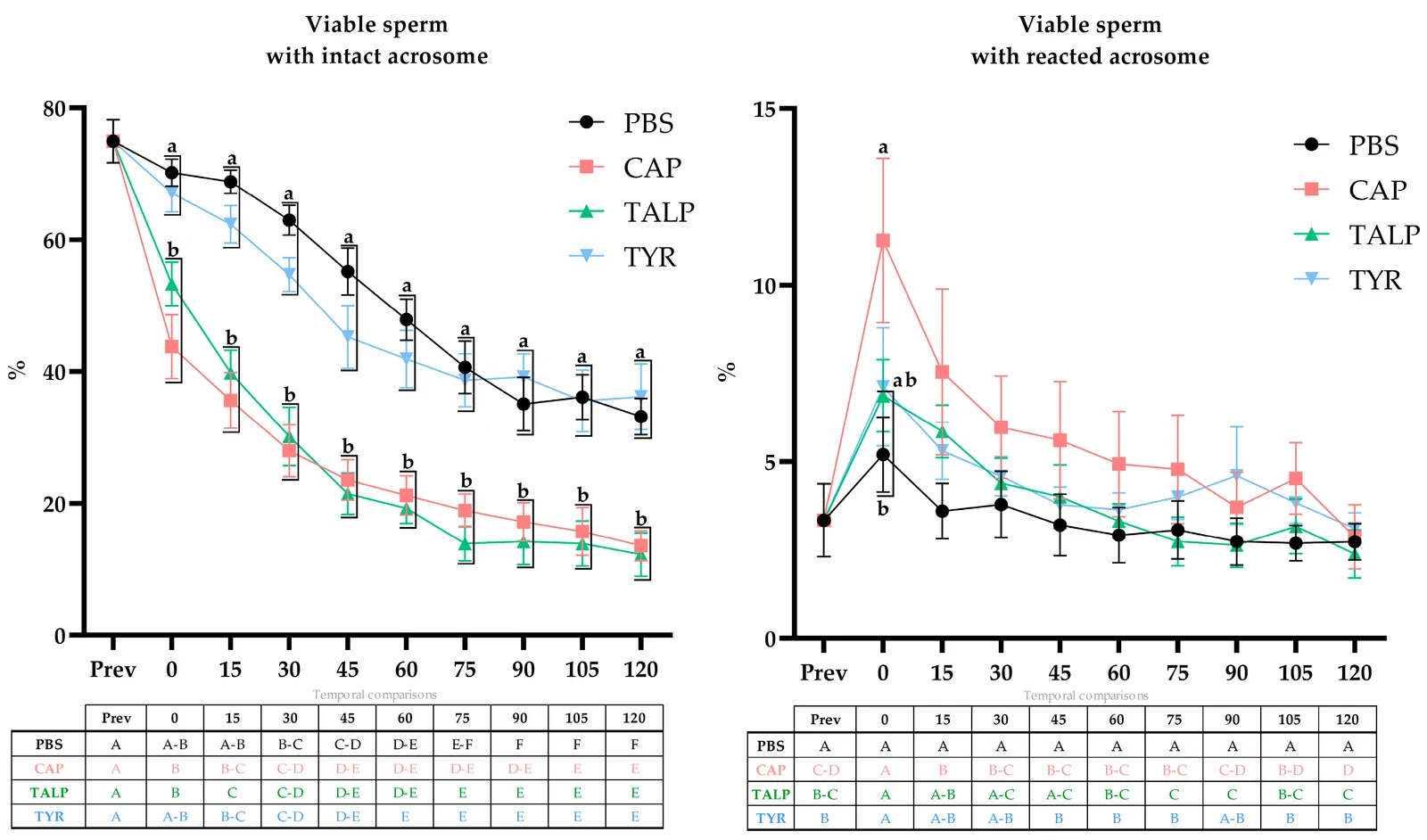
Evaluation of the viability and acrosome integrity of different capacitation extenders over time. PBS: phosphate-buffered saline (Sigma-Aldrich), non-capacitating control. CAP: modified version of Brackett and Oliphant, 1975 extender. TALP: modified version of Tyrode’s extender. TYR: a classical Tyrode’s formulation. Viable sperm with intact acrosome (Yop–/PNA–); viable sperm with reacted acrosome (Yop–/PNA+). Lowercase letters above the curves indicate significant differences among treatments at each incubation time (*p* < 0.05). Uppercase letters in the table below each graph indicate significant differences among incubation times within the same treatment (*p* < 0.05). Treatments or time points sharing at least one letter are not significantly different. For visual clarity, multiple consecutive letters sharing non-significant differences are presented as ranges (e.g., A–D). Data are presented as mean ± standard error of the mean (SEM; *n* = 8 pools).

The proportion of viable sperm with intact acrosomes (Yop–/PNA–) was significantly lower (*p* < 0.05) in the CAP and TALP media compared to TYR and PBS throughout the whole incubation time (Figure 1).

Furthermore, both media—especially CAP—caused an immediate and significant increase in reacted acrosomes (Yop–/PNA+) at the time of dilution (0 min), reaching similar values to the other treatments at 15 min and maintaining these values throughout the incubation period. Notably, PBS was the only treatment that did not show significant differences over time for this viable subpopulation with reacted acrosomes, remaining completely stable throughout the 120 min. Moreover, both populations of non-viable sperm with intact acrosomes (Yop+/PNA–) and reacted acrosomes (Yop+/PNA+) (Table 2) were significantly higher in the CAP and TALP treatments and increased faster starting at the earliest incubation times.

**Figure 2:**
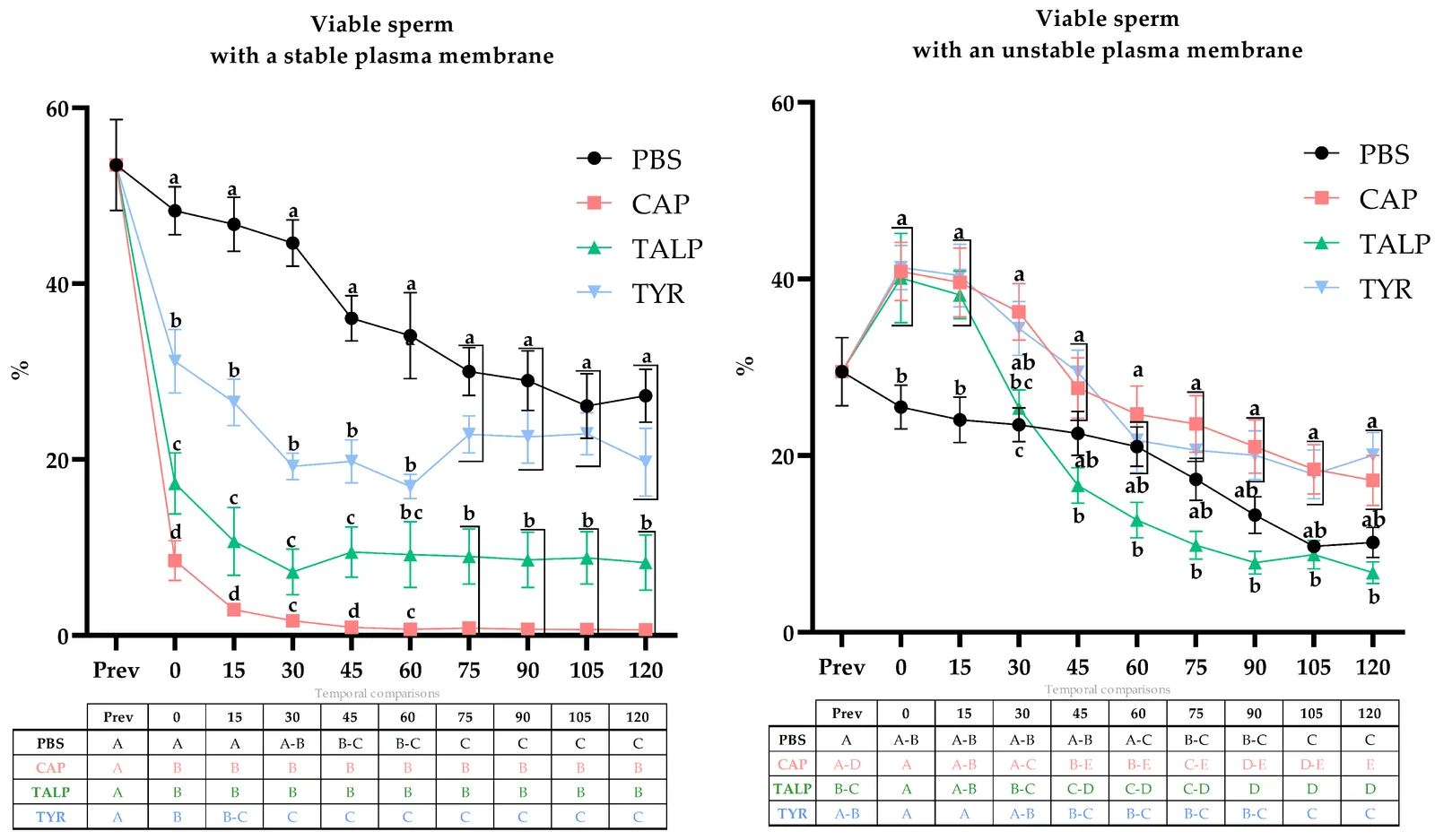
Evaluation of the viability and membrane lipid disorder of different capacitation extenders over time. PBS: phosphate-buffered saline (Sigma-Aldrich), non-capacitating control. CAP: modified version of Brackett and Oliphant, 1975 extender. TALP: modified version of Tyrode’s extender. TYR: a classical Tyrode’s formulation. Viable sperm with a stable plasma membrane (Yop–/M540–); Viable sperm with an unstable plasma membrane (Yop–/M540+). Lowercase letters above the curves indicate significant differences among treatments at each incubation time (*p* < 0.05). Uppercase letters in the table below each graph indicate significant differences among incubation times within the same treatment (*p* < 0.05). Treatments or time points sharing at least one letter are not significantly different. For visual clarity, multiple consecutive letters sharing non-significant differences are presented as ranges (e.g., A–D). Data are presented as mean ± standard error of the mean (SEM; *n* = 8 pools).

In the membrane lipid disorder, measured using the M540 probe, our results showed that, in the first minutes (0 and 15 min), the three capacitation media significantly increased the population of viable sperm with an unstable membrane (Yop–/M540+) (Figure 2).

PBS also exhibited a high initial percentage of viable sperm with a lipid membrane disorder, although this response remained lower than that of the capacitating media during the initial incubation period. The population of viable sperm with stable lipid organization (Yop–/M540–) significantly decreased in all media, especially TALP and CAP. Despite this decline, TYR was the only medium that maintained this population at relatively higher and more stable levels over time (Figure 2) without differences with the PBS after one hour of incubation. Furthermore, the proportion of non-viable sperm with unstable membranes (Yop+/M540+) increased rapidly and steadily in CAP and TALP, reaching significantly higher values than in PBS and TYR (*p* < 0.05) (Table 2).

**Figure 3:**
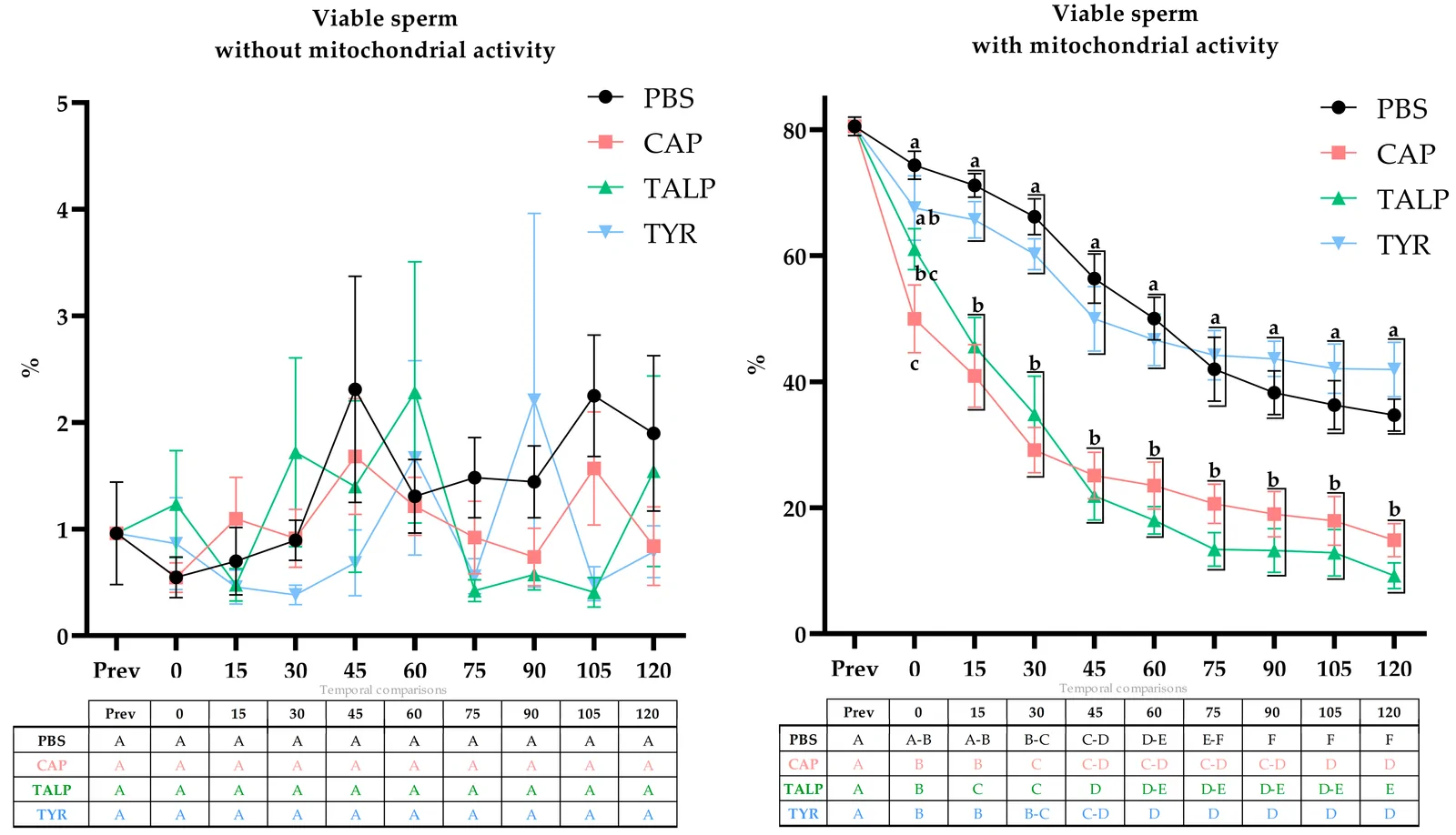
Evaluation of the viability and mitochondrial activity of different capacitation extenders over time. PBS: phosphate-buffered saline (Sigma-Aldrich), non-capacitating control. CAP: modified version of Brackett and Oliphant, 1975 extender. TALP: modified version of Tyrode’s extender. TYR: a classical Tyrode’s formulation. Viable sperm without mitochondrial activity (Yop–/MTdr–); Viable sperm with mitochondrial activity (Yop–/MTdr+). Lowercase letters above the curves indicate significant differences among treatments at each incubation time (*p* < 0.05). Uppercase letters in the table below each graph indicate significant differences among incubation times within the same treatment (*p* < 0.05). Treatments or time points sharing at least one letter are not significantly different. For visual clarity, multiple consecutive letters sharing non-significant differences are presented as ranges (e.g., A–D). Data are presented as mean ± standard error of the mean (SEM; *n* = 8 pools).

Regarding mitochondrial activity and viability, CAP and TALP treatments showed a lower percentage of viable sperm with high mitochondrial activity (Yop–/MTdr+) than TYR and PBS, with similar results for non-viable sperm without mitochondrial activity (Yop+/MTdr–) (Figure 3 and Table 2).

Non-viable sperm with mitochondrial activity (Yop+/MTdr+) were significantly more abundant in the CAP treatment than in the other treatments, especially during the first 60 min of incubation.

Lastly, sperm motility and kinetic analyses also showed clear differences between the different capacitation media (Table 3).

In terms of overall motility, no significant differences were observed immediately after the medium change. Moreover, no significant differences were found between the media and the PBS control. However, a significant decrease in TM was observed at 60 min in the CAP and TALP treatments, while the TYR medium maintained significantly higher values, although not different from PBS. The PM results showed a similar pattern to the TM. In contrast, the kinetic parameters exhibited distinct trends among media (Table 3). At the beginning of incubation (0 min), PBS showed the highest curvilinear velocity (VCL), significantly higher than TALP and CAP (*p* < 0.05), while TYR presented intermediate values. After 60 min, PBS still maintained the highest VCL, with TYR and CAP showing intermediate values and TALP remaining the lowest. However, for VSL and VAP, although PBS and TYR showed higher values than CAP and TALP, these results converged over time. Regarding movement patterns, TYR promoted higher LIN, STR and WOB values compared to the other media, both at 60 and 120 min (*p* < 0.05), and was different from PBS only in LIN.

## 4. Discussion

Sperm in vitro capacitation remains non-standardized due to different capacitation media used in the laboratories. The different procedures employed, including the capacitation media, make it difficult to compare results obtained between different laboratories. Indeed, our results show important differences in the sperm response to the different capacitation media used. One of the most remarkable findings is the abrupt increase in viable spermatozoa showing reacted acrosomes and membrane lipid disorder immediately at time 0, especially in the CAP medium. These changes appeared immediately after centrifugation and media exchange. We propose as a plausible biological hypothesis that spermatozoa may already have been partially sensitized before the incubation period itself, which could explain why spermatozoa already displayed a high proportion of viable cells with membrane lipid disorder before the capacitation treatments were initiated.

Interestingly, the membrane lipid disorder in CAP and TALP remained elevated during the first 30–45 min, whereas TYR showed a more gradual and sustained response over time. However, it should be taken into account that the PBS treatment also showed relatively high values for these parameters. These changes are unlikely to be caused by PBS itself, but rather reflect the physiological state of the sperm population before the medium exchange. As a potential explanation, we hypothesize that the capacitation process may be influenced not only by the incubation medium but also by plausible pre-capacitation handling mechanisms, such as centrifugation, the removal of the storage extender and seminal plasma, or even the previous incubation of spermatozoa in semen preservation media [26,27,28].

Some studies have reported that incubations longer than 60 min do not increase the percentage of capacitated spermatozoa [9,29,30], and may even be detrimental. However, several other studies indicate that the highest proportion of sperm showing capacitation-associated changes can occur after more than two hours of incubation [26,31,32,33]. This marked discrepancy with our results highlights the current challenge in defining and standardizing an in vitro capacitation protocol in boar spermatozoa. Multiple factors could affect the capacitation-associated response, such as the type of refrigeration or capacitation medium used, possible modifications of the composition and concentrations, and the origin of the sperm sample itself. Indeed, some studies have used diluted semen [7,26,32], frozen–thawed semen [6,34,35], or even epididymal samples [9]. These differences may explain why similar media often lead to divergent outcomes.

Therefore, under our experimental conditions, it becomes important to consider why spermatozoa responded so rapidly to the capacitating media and why the magnitude of this response differed among media. As previously mentioned, most studies do not standardize several preliminary steps that can be crucial, such as the storage extender used before capacitation, the duration of refrigeration, the centrifugation force applied to remove the storage extender and the seminal plasma. While these pre-capacitation factors were not directly tested in our experimental setup, they represent plausible mechanisms that could influence capacitation outcomes and partly explain the differences observed between our results and those reported by other authors. This could provide insight into the results observed in our non-capacitating control. In the PBS treatment, several sperm subpopulations displayed patterns that partially resembled those observed in the capacitation media, particularly during the earliest incubation times. However, these values most likely reflect the physiological condition already present before incubation, since the composition of PBS is not expected to induce or amplify these responses [24]. In fact, these parameters gradually decreased over time compared with the values recorded immediately after the pre-capacitation step. This could suggest that some of the sperm population was already in a destabilized or sensitized state before incubation, likely as a consequence of the aforementioned factors. In this context, the capacitation media might not necessarily initiate all the observed changes de novo, but rather amplify or accelerate membrane and acrosome responses that had already begun in a proportion of the sperm.

This hypothesis centers on the type of refrigeration extender and the duration of semen storage at 15–17 °C. Spermatozoa can alter their responsiveness to bicarbonate the longer they remain under refrigeration [21,28,34,36,37,38]. This could be consistent with our findings, since the semen doses used in our experiments were stored for more than 24 h at 17 °C, with the most pronounced membrane destabilization and acrosomal response in the capacitation medium containing the highest bicarbonate concentration (CAP). Another important factor is the type of cooling extender used before capacitation [27,38,39]. It has been shown that sperm diluted in short-term extenders such as BTS progressively lose their ability to respond to capacitating stimuli over time [38]. In contrast, some extenders may preserve sperm functionality better, allowing stronger capacitation responses, whereas others reduce sensitivity to bicarbonate and albumin [27]. The preservation extender used in our semen doses was a long-term extender (Duragen, Magapor), yet our results show that spermatozoa already exhibited a higher initial degree of lipid disorder, which might be linked to the earlier capacitation response observed in this study. This initial lipid disorder has also been reported in other studies using the same storage extender [32]. However, a major difference is that in their case, semen was stored for only 45 min, much shorter than in our study. In addition, they used the TYR medium for capacitation, with modifications such as higher BSA and calcium concentrations, which could explain the distinct capacitation behavior reported by those authors. Moreover, those studies and others [26,40] used much lower centrifugation forces (between 300 and 600× *g*) compared with ours (1500× *g*), which may provide another possible explanation for the differences between studies. It has been demonstrated that centrifugation can modify the sperm response to bicarbonate [28], a key stimulus for inducing capacitation, and that this effect is even more pronounced in semen stored for more than 12 h at 17 °C [21,28,34,36,37,38]. This agrees with our results since we used longer storage times and higher centrifugation speeds. Regarding centrifugation, another plausible mechanism is that removing seminal plasma (along with the storage extender) also eliminates extracellular vesicles (EVs). Xu et al. (2024) [41] observed that EVs inhibit the acrosome reaction; therefore, their removal during centrifugation might facilitate the acrosome reaction observed at time 0 in media containing high levels of bicarbonate and caffeine (CAP medium). In addition, Du et al. (2016) [42] reported that these vesicles integrate into the sperm membrane, and the longer they remain in contact, the greater their incorporation. Thus, the storage time at 15–17 °C could influence this process and, consequently, the sperm sensitivity to capacitation, potentially requiring higher concentrations of capacitating compounds such as those present in the CAP medium.

Sperm motility, on the other hand, exhibited a distinct pattern, likely due to the combined effects of seminal plasma removal and subsequent re-dilution, together with the specific composition of the media used for sperm resuspension. Accordingly, no significant differences in total motility (TM) or progressive motility (PM) were observed among treatments at time 0, whereas significant differences were detected in velocity parameters and sperm movement patterns. Interestingly, PBS showed significantly higher velocity values at time 0 compared with the other treatments. This finding may be largely attributed to the absence of bovine serum albumin (BSA), resulting in a less viscous medium that allows greater sperm swimming velocity [43]. In contrast, the reduction in velocity observed in CAP and TALP (and to a lesser extent in TYR) at time 0 may be explained by the immediate synergistic action of BSA and the different bicarbonate concentrations present in each medium. Bicarbonate rapidly stimulates the soluble adenylyl cyclase (sAC) pathway, altering membrane fluidity and facilitating the prompt extraction of cholesterol from the plasma membrane by BSA [32]. This immediate loss of membrane cholesterol modifies the permeability of ion channels, thereby affecting both the sperm motility pattern and velocity parameters [7]. This mechanism may explain the greater reduction in velocity observed in media containing higher bicarbonate concentrations.

Furthermore, bicarbonate may be one of the main factors responsible for the rapid changes observed in acrosomal status and membrane fluidity. Although its precise role remains a matter of debate, bicarbonate has been consistently recognized as a key regulator of sperm capacitation. Upon entering the sperm cell, bicarbonate activates the cAMP/PKA and Ca^2+^ signaling pathways, which are essential for membrane remodeling and the acquisition of the capacitated state [41]. It also activates enzymes that translocate membrane phospholipids, such as phosphatidylserine and phosphatidylethanolamine, thereby reducing membrane stability and making cholesterol available for external acceptors like BSA [41,44,45]. However, its effect appears to be strongly concentration-dependent. Some studies suggest that moderate bicarbonate concentrations are sufficient to induce capacitation-associated changes while preserving sperm functionality and reducing polyspermy rates [11,30]. In contrast, higher concentrations may accelerate membrane destabilization and acrosomal exocytosis, but at the expense of sperm viability and membrane integrity [9]. This seems consistent with our observations, where CAP induced a rapid and intense response that was less sustainable over time, whereas TYR maintained sperm functionality more consistently during incubation.

Caffeine may also contribute to the observed effects, as it is a well-known phosphodiesterase inhibitor that increases intracellular cAMP levels and can modulate both motility and the acrosome reaction in spermatozoa [17]. Indeed, several authors have reported that high caffeine concentrations can be detrimental, leading to excessive acrosomal reactivity [17,44,46]. This could explain the higher proportion of viable sperm with reacted acrosomes, and of non-viable sperm showing acrosomal exocytosis in treatments with higher caffeine content (CAP and TALP) compared to those without caffeine (TYR and PBS). However, caffeine does not appear to have a direct effect on the percentage of motile sperm [7], as the medium without caffeine maintained motility and movement quality better over time.

Sperm motility may also be largely influenced by the availability and type of energy substrates. Boar spermatozoa can obtain energy through two main metabolic pathways [45,47]: glycolysis, which occurs in the cytoplasm using glucose as a substrate, and oxidative phosphorylation (OXPHOS), which takes place in mitochondria and depends on pyruvate as an intermediate. Although these pathways are interconnected, their differential activation can have distinct functional consequences. The glycolytic pathway produces ATP more quickly and in the principal piece, but it is less efficient. In contrast, lactate and pyruvate favor OXPHOS [45,47], generating ATP more efficiently and sustainably, but increasing intracellular ROS [48]. Serrano et al. (2025) and Amaral (2022) [31,48] reported that glucose may have a detrimental effect on boar sperm capacitation, particularly at higher concentrations. These findings suggest that boar spermatozoa may perform more efficiently in media that promote oxidative phosphorylation rather than glycolysis [49]. Evidence for this can be observed in the present study, where PBS, despite lacking exogenous energy substrates, maintained sperm motility over time more effectively than CAP and TALP, although slightly less efficiently than TYR. In the absence of external substrates, spermatozoa likely rely on endogenous lipid reserves and intracellular lactate/pyruvate pools as substrates for mitochondrial oxidation [50]. TYR, in contrast, contains higher concentrations of lactate and pyruvate than the other media, which may enhance the intracellular availability of these substrates without shifting sperm metabolism toward glycolysis, owing to its relatively lower glucose content. Consequently, TYR may support mitochondrial ATP production while avoiding the potentially negative effects associated with increased glycolytic activity. This metabolic profile could explain why, despite inducing membrane changes comparable to those observed in the other capacitating media, TYR was more effective at maintaining sperm motility over time. Furthermore, TYR produced significantly higher LIN, STR, and WOB values than CAP and TALP, although statistical significance relative to PBS was only observed for LIN. This more linear trajectory was described by [29], using a medium of nearly identical composition, as characteristic of the capacitated state in porcine spermatozoa.

Several limitations should be considered when interpreting these findings. First, the capacitation media differed simultaneously in several components; therefore, the contribution of individual components cannot be established from the present experimental design. Second, semen was obtained from commercially stored doses and subjected to centrifugation before incubation, meaning that storage and processing may have influenced the initial physiological state of spermatozoa. Third, while flow cytometry provided high-temporal-resolution kinetics every 15 min, CASA evaluations were limited to 0, 60, and 120 min due to the technical constraint of simultaneously handling and processing all four treatments across tightly timed cytometry protocols. Consequently, subtle motility shifts occurring within the first hour may have gone undetected between measured time points. Finally, functional fertilizing capacity (e.g., IVF outcomes or zona pellucida binding) was not evaluated. Therefore, the mechanistic interpretations proposed here should be considered hypotheses that warrant targeted experimental validation, where incorporating IVF assays and specific metabolic markers will be essential to directly link these kinetic phenotypes to fertilizing potential.

## 5. Conclusions

Therefore, the composition of capacitation media appears to influence the timing and magnitude of several capacitation-associated sperm responses in boar spermatozoa. Under our conditions, extenders containing higher bicarbonate and caffeine concentrations promoted a rapid membrane and acrosomal response, although this effect was accompanied by a faster decline in sperm viability over time. In contrast, TYR maintained sperm functionality and more sustained motility patterns during incubation. Overall, these findings indicate that the outcome of in vitro capacitation is determined not only by the composition of the incubation medium but also by the physiological status of spermatozoa before capacitation, which is influenced by semen storage and handling. Altogether, these insights could help improve the future standardization of porcine sperm capacitation protocols, although incorporating functional fertilizing assays such as IVF will be necessary to fully validate these findings.

## Figures and Tables

**Table 1:** Capacitation extenders composition.

Concentration (mM)	CAP	TALP	TYR
NaCl	112	114	96
KCl	4.02	3.2	3.1
Na_2_HPO_4_	0.83	0.4	-
MgCl_2_ 6H_2_O	0.52	0.5	0.4
Glucose	13.9	5	5
Sodium Pyruvate	1.25	0.1	1
Sodium Lactate	-	10	21.7
Hepes	-	-	20
NaHCO_3_	37	25	15
CaCl_2_	2.25	2	2
BSA (mg/mL)	3	3	3
Caffeine	5.15	2	-
pH	7.8	7.4	7.44
Osmolality (mOsm/kg)	309	286	270

CAP: modified version of Brackett and Oliphant, 1975 extender. TALP: modified version of Tyrode’s extender. TYR: a classical Tyrode’s formulation. BSA: bovine serum albumin.

**Table 2:** Results of non-viable boar sperm subpopulations assessed by flow cytometry during in vitro incubation in different capacitation media.

	Treatments	Prev	0 min	15 min	30 min	45 min	60 min	75 min	90 min	105 min	120 min
Yop+/PNA–	PBS	5.5 ± 0.8	4.4 ± 1.5 ^c^	8.6 ± 1.92 ^c^	9.3 ± 0.8 ^b^	12.1 ± 1.9 ^b^	15.1 ± 2.2 ^b^	15.5 ± 1.7 ^ab^	16.9 ± 2.2 ^ab^	17.3 ± 3.2	13.7 ± 2.8
	CAP	5.5 ± 0.8 ^3^	11.6 ± 3.4 ^a;2–3^	20.6 ± 3.3 ^a;1–2^	27 ± 3.6 ^a;1^	27.7 ± 3.2 ^a;1^	28.4 ± 4.2 ^a;1^	23.7 ± 4.2 ^ab;1–2^	23.5 ± 3.6 ^ab;1–2^	21.3 ± 4.5 ^1–2^	21.7 ± 5.2 ^1–2^
	TALP	5.5 ± 0.8 ^4^	10.2 ± 3.1 ^ab;3–4^	14.9 ± 2.7 ^b;2–4^	23.4 ± 5.3 ^a;1–2^	25.8 ± 5.8 ^a;1^	30 ± 6.7 ^a;1^	24.7 ± 5.4 ^a;1–2^	22.5 ± 4.9 ^ab;1–3^	19.5 ± 6 ^1–3^	19.4 ± 5.8 ^1–3^
	TYR	5.5 ± 0.8 ^2^	6.6 ± 2.1 ^bc;2^	9.5 ± 1.7 ^c;1–2^	13.8 ± 2.1 ^b;1–2^	16.6 ± 2.7 ^b;1–2^	21.1 ± 5 ^ab;1^	15 ± 2.3 ^b;1–2^	15.9 ± 1.9 ^b;1–2^	14.4 ± 2.6 ^1–2^	18.2 ± 4.4 ^1–2^
Yop+/PNA+	PBS	16.2 ± 2.5 ^1^	20.2 ± 1.4 ^cb;1–2^	19 ± 1.3 ^b;1–2^	23.9 ± 1.8 ^b;1–2^	29.55 ± 2.6 ^c;1–3^	34.2 ± 2.7 ^b;2–4^	40.8 ± 4.1 ^b;3–5^	45.3 ± 5.4 ^bc;4–5^	43.8 ± 3.8 ^c;4–5^	50.4 ± 4.6 ^ab;5^
	CAP	16.2 ± 2.5 ^1^	34.8 ± 3.7 ^a;1–2^	36.2 ± 3.9 ^a;2–3^	39.1 ± 4.4 ^a;2–4^	43.1 ± 4.1 ^ab;3–5^	40.8 ± 5.2 ^a;4–6^	52.6 ± 5.3 ^a;5–7^	55.6 ± 5.4 ^ab;6–7^	58.5 ± 7.1 ^ab;7^	61.9 ± 6.4 ^a;7^
	TALP	16.2 ± 2.5 ^1^	29.6 ± 3.7 ^ab;2^	39.4 ± 4.2 ^a;2^	42 ± 5 ^a;2–3^	48.8 ± 4.4 ^a;2–4^	47.4 ± 5.5 ^a;2–5^	58.7 ± 5.1 ^a;3–6^	60.6 ± 5.7 ^a;4–6^	63.4 ± 6.6 ^a;5–6^	66 ± 6.2 ^a;6^
	TYR	16.2 ± 2.5 ^1^	19.2 ± 1.8 ^b;1–3^	22.9 ± 2.3 ^b;1–3^	26.9 ± 2.5 ^b;1–3^	34.4 ± 3.8 ^bc;2–4^	33.3 ± 5.1 ^b;3–5^	42.4 ± 5.1 ^b;4–5^	40.3 ± 4.7 ^c;5^	46.2 ± 5.6 ^bc;5^	42.5 ± 6.4 ^b;5^
Yop+/M540–	PBS	3.89 ± 0.6	5 ± 0.6 ^a^	5 ± 1.3 ^ab^	3.9 ± 0.6 ^ab^	3.3 ± 0.3 ^ab^	3.3 ± 0.7	5.7 ± 0.6	6.2 ± 1.2	5.4 ± 0.9	6 ± 1.7
	CAP	3.89 ± 0.6	5.6 ± 0.6 ^ab^	5.6 ± 1.2 ^a^	5.9 ± 0.9 ^a^	3.4 ± 0.6 ^ab^	3.4 ± 0.8	4.2 ± 1.1	3.4 ± 0.5	3.4 ± 0.7	4.3 ± 0.9
	TALP	3.89 ± 0.6	2.6 ± 0.2 ^b^	2.6 ± 0.1 ^b^	3.2 ± 0.4 ^b^	4.8 ± 1.3 ^a^	3.6 ± 0.9	4.5 ± 1.1	5.6 ± 2.5	5.2 ± 1.8	5.4 ± 2
	TYR	3.89 ± 0.6	2.3 ± 0.4 ^b^	2.3 ± 0.4 ^b^	2.3 ± 0.3 ^b^	2.5 ± 0.5 ^b^	2.3 ± 0.4	4.1 ± 0.7	3.7 ± 0.6	3.6 ± 0.7	3.6 ± 0.9
Yop+/M540+	PBS	13.1 ± 2.1 ^1^	20.8 ± 1.8 ^b;1–2^	24.2 ± 2.1 ^b;1–2^	27.9 ± 1.8 ^c;2–3^	38.1 ± 4.1 ^b;3–4^	41.4 ± 5.4 ^b;4–5^	47 ± 3.6 ^b;5–7^	51.6 ± 4.5 ^b;5–7^	58.8 ± 4.3 ^b;7^	56.6 ± 4.2 ^b;6–7^
	CAP	13.1 ± 2.1 ^1^	46.7 ± 4.7 ^a;2^	51.8 ± 4.2 ^a;2–3^	56.1 ± 3.1 ^a;3–4^	68 ± 3.6 ^a;3–5^	69.7 ± 3.7 ^a;4–5^	71.4 ± 4 ^a;4–5^	74.9 ± 3.4 ^a;5^	77.5 ± 3.3 ^a;5^	77.9 ± 3.5 ^a;5^
	TALP	13.1 ± 2.1 ^1^	41 ± 3.6 ^a;2^	48.5 ± 3.8 ^a;2^	64.2 ± 4.2 ^a;3^	69 ± 3.8 ^a;3–4^	74.1 ± 4.5 ^a;3–4^	76.7 ± 4.2 ^a;4^	77.9 ± 4.4 ^a;4^	77.2 ± 4.4 ^a;4^	79.6 ± 4.3 ^a;4^
	TYR	13.1 ± 2.1 ^1^	25.7 ± 3.1 ^b;2^	31.1 ± 2 ^b;2–3^	44 ± 3.4 ^b;3–4^	48.2 ± 3.5 ^b;4–5^	58.4 ± 4.5 ^a;4–5^	52.4 ± 3.6 ^b;4–5^	56.7 ± 4.4 ^b;5^	55.6 ± 4.5 ^b;5^	56.6 ± 4.2 ^b;5^
Yop+/MTdr–	PBS	8 ± 1.5 ^1^	14.6 ± 1.9 ^c;1^	15.94 ± 2 ^b;1^	20.3 ± 2.5 ^b;1–2^	29.6 ± 3.2 ^c;2–3^	36.8 ± 4.2 ^c;2–3^	44.6 ± 4.5 ^c;4–5^	47.2 ± 3.7 ^b;4–5^	51.7 ± 4.3 ^bc;5^	53.3 ± 2.3 ^b;5^
	CAP	8 ± 1.5 ^1^	33.3 ± 4.1 ^a;2^	38.1 ± 6.2 ^a;2–3^	46.4 ± 6.9 ^a;3–4^	50.2 ± 6.1 ^ab;3–5^	53.4 ± 6.9 ^a;4–6^	60.4 ± 6.15 ^b;5–7^	64.7 ± 5.6 ^a;6–7^	63.2 ± 7.6 ^ab;7^	69.7 ± 3 ^a;7^
	TALP	8 ± 1.5 ^1^	24.4 ± 2.5 ^ab;2^	39.3 ± 2.4 ^a;3^	50.1 ± 5.8 ^a;3^	64.2 ± 3.5 ^a;4^	66.2 ± 2.8 ^a;4–5^	77.8 ± 4.6 ^a;5–6^	76.8 ± 5.5 ^a;5–6^	77.8 ± 5.3 ^a;5–6^	79.9 ± 4.5 ^a;6^
	TYR	8 ± 1.5 ^1^	19.6 ± 4.6 ^bc;1–2^	21.4 ± 3 ^b;2^	25.9 ± 3.5 ^b;2–3^	36.6 ± 3.6 ^bc;3–4^	37.5 ± 4.3 ^b;3–4^	42.4 ± 4.5 ^c;4^	42.6 ± 3.4 ^b;4^	44.9 ± 2.7 ^c;4^	45.5 ± 4 ^b;4^
Yop+/MTdr+	PBS	10.5 ± 1.4	10.5 ± 2 ^b^	12.2 ± 3.1 ^b^	12.6 ± 2.7 ^b^	11.7 ± 3 ^b^	11.9 ± 3.3 ^b^	11.9 ± 3.1 ^ab^	13.1 ± 4.1	9.7 ± 2.3 ^ab^	9.85 ± 1.4 ^b^
	CAP	10.5 ± 1.4 ^3^	16.7 ± 2.6 ^a;1–3^	19.9 ± 3.8 ^a;1–2^	23.5 ± 5.6 ^a;1^	23 ± 5.6 ^a;1–2^	21.9 ± 5.3 ^a;1–2^	18 ± 4.3 ^a;1–3^	15.6 ± 3.5 ^1–3^	17.3 ± 5 ^a;1–3^	14.6 ± 2.5 ^a;2–3^
	TALP	10.5 ± 1.4	13.3 ± 2.1 ^ab^	14.6 ± 2.5 ^a-b^	13.3 ± 2.6 ^b^	12.5 ± 2.3 ^a^	13.5 ± 3 ^ab^	8.4 ± 2.1 ^b^	9.4 ± 2.8	8.9 ± 2.2 ^b^	9.4 ± 2.5 ^b^
	TYR	10.5 ± 1.4	11.9 ± 1.9 ^ab^	12.4 ± 1.9 ^b^	13.5 ± 2.7 ^b^	12.8 ± 2.5 ^a^	14.2 ± 3.1 ^ab^	12.8 ± 3.1 ^ab^	11.5 ± 2.1	12.5 ± 2.5 ^ab^	11.7 ± 2 ^ab^

PBS: phosphate-buffered saline (Sigma-Aldrich), non-capacitating control. CAP: modified version of Brackett and Oliphant, 1975 medium. TALP: modified version of Tyrode’s medium. TYR: a classical Tyrode’s formulation. Non-viable sperm populations (exhibiting compromised plasma membranes) were identified across all three multiplexed fluorochrome assays by positive staining for YoPro-1 (Yop+). Subpopulations are defined as follows: (i) Acrosomal integrity assay: Yop+/PNA– (non-viable sperm with intact acrosome) and Yop+/PNA+ (non-viable sperm with reacted acrosome); (ii) Membrane lipid disorder assay: Yop+/M540– (non-viable sperm with stable lipid architecture) and Yop+/M540+ (non-viable sperm with high membrane lipid disorder); (iii) Mitochondrial potential assay: Yop+/MTdr– (non-viable sperm without mitochondrial activity) and Yop+/MTdr+ (non-viable sperm retaining active mitochondrial potential). Different superscript letters (a, b, c) within the same row indicate significant differences among treatments within each specific incubation time (*p* < 0.05). Different superscript numbers indicate significant differences between time points within the same treatment (*p* < 0.05). Superscript letters and numbers are separated by a semicolon (e.g., a;1–3), where hyphens represent ranges of time points sharing non-significant differences. Data are shown as mean ± standard error of the mean (SEM; *n* = 8 pools).

**Table 3:** Motility and kinematic parameters during in vitro capacitation with different capacitation extenders after 120 min of incubation.

	Treatments	Prev	0 min	60 min	120 min
Total Motility (TM, %)	PBS	76.3 ± 5.5	78.6 ± 3.5	63.9 ± 5.9 ^ab^	65.1 ± 4.5 ^ab^
	CAP	76.3 ± 5.5 ^1^	71.4 ± 4.9 ^1^	51.8 ± 3.8 ^b;2–3^	47.6 ± 4.3 ^b;3^
	TALP	76.3 ± 5.5 ^1^	66.8 ± 4.6 ^1^	47.4 ± 5.2 ^b;1–2^	49.8 ± 7 ^b;2^
	TYR	76.3 ± 5.5	83.7 ± 3	73.7 ± 5.7 ^a^	71.7 ± 6.1 ^a^
Progressive Motility (PM, %)	PBS	37.7 ± 2.5	50.2 ± 3.1	42.8 ± 4.7 ^ab^	44.5 ± 3.3 ^ab^
	CAP	37.7 ± 2.5 ^1–2^	48.7 ± 3.9 ^1^	31.3 ± 2.9 ^b;2^	29.2 ± 3.8 ^c;2^
	TALP	37.7 ± 2.5 ^1–2^	45.2 ± 3.8 ^1^	29.6 ± 3.5 ^b;2^	31.5 ± 4.9 ^bc;1–2^
	TYR	37.7 ± 2.5 ^2^	56.3 ± 2.1 ^1^	51.9 ± 4.3 ^a;1–2^	50.9 ± 4.6 ^a;1–2^
Curvilinear Velocity (VCL, µm/s)	PBS	169.7 ± 12.2 ^1^	209.8 ± 10.3 ^a;2^	174.9 ± 4.9 ^a;1–2^	163.5 ± 7.9 ^1^
	CAP	169.7 ± 12.2	153.2 ± 6.3 ^bc^	153.5 ± 5.5 ^ab^	150.2 ± 3.8
	TALP	169.7 ± 12.2	138 ± 3.8 ^c^	144.5 ± 3.6 ^b^	147.7 ± 4.4
	TYR	169.7 ± 12.2	175.1 ± 10.4 ^b^	153.7 ± 11 ^ab^	145 ± 5.7
Straight-line Velocity (VSL, µm/s)	PBS	36.4 ± 2.1 ^3^	69.1 ± 6.3 ^a;1^	53.4 ± 3.6 ^a;2^	51.6 ± 4.5 ^a;2^
	CAP	36.4 ± 2.1	45.9 ± 3.2 ^b^	40.9 ± 2 ^ab^	39.7 ± 1.7 ^b^
	TALP	36.4 ± 2.1	41 ± 1.8 ^b^	38.1 ± 1.9 ^b^	39.1 ± 1.7 ^b^
	TYR	36.4 ± 2.1 ^2^	58 ± 5 ^ab;1^	53 ± 5.6 ^a;1^	50.5 ± 3.2 ^ab;1–2^
Average path Velocity (VAP, µm/s)	PBS	76.1 ± 5.9 ^1^	107.1 ± 7.4 ^a;2^	89.6 ± 2.9 ^a;1–2^	85.2 ± 4.4 ^1^
	CAP	76.1 ± 5.9	76.9 ± 3.7 ^b^	75.2 ± 3 ^ab^	75.4 ± 1.9
	TALP	76.1 ± 5.9	71.1 ± 1.6 ^b^	72.4 ± 1.5 ^b^	74.7 ± 3.2
	TYR	76.1 ± 5.9	90.6 ± 6.2 ^ab^	82.3 ± 6.6 ^ab^	78.4 ± 3.4
Linearity (LIN, %)	PBS	24.9 ± 1.2 ^2^	29.1 ± 1.1 ^1–2^	29.5 ± 1.1 ^b;1–2^	30.5 ± 0.8 ^b;1^
	CAP	24.9 ± 1.2 ^2^	29.7 ± 1.2 ^1^	27.6 ± 1 ^b;1–2^	28.1 ± 1.1 ^b;1–2^
	TALP	24.9 ± 1.2 ^2^	31 ± 1.2 ^1^	28.9 ± 0.5 ^b;1–2^	28.6 ± 1.3 ^b;1–2^
	TYR	24.9 ± 1.2 ^2^	31.5 ± 1 ^1^	34.4 ± 1 ^a;1^	34.9 ± 1 ^a;1^
Straightness (STR, %)	PBS	52 ± 2.3 ^2^	58.1 ± 0.9 ^1–2^	59.5 ± 1.1 ^ab;1^	60.7 ± 0.9 ^ab;1^
	CAP	52 ± 2.3 ^2^	61 ± 0.8 ^1^	55.9 ± 1.3 ^b;1–2^	55.9 ± 1.7 ^b;1–2^
	TALP	52 ± 2.3 ^2^	60.8 ± 1.3 ^1^	57.4 ± 0.9 ^b;1–2^	56.9 ± 2.1 ^b;1–2^
	TYR	52 ± 2.3 ^2^	61 ± 0.7 ^1^	63.3 ± 1 ^a;1^	63.8 ± 0.9 ^a;1^
Wobble coefficient (WOB, %)	PBS	44.8 ± 1 ^2^	50.7 ± 1.1 ^1^	51.2 ± 0.8 ^ab;1^	52.1 ± 0.4 ^ab;1^
	CAP	44.8 ± 1 ^2^	50.2 ± 1 ^1^	48.9 ± 0.5 ^b;1^	50.2 ± 0.3 ^b;1^
	TALP	44.8 ± 1 ^2^	51.7 ± 1 ^1^	50.2 ± 0.7 ^b;1^	50.5 ± 1.1 ^b;1^
	TYR	44.8 ± 1 ^2^	51.6 ± 0.9 ^1^	53.4 ± 0.8 ^a;1^	54 ± 0.7 ^a;1^

PBS: phosphate-buffered saline (Sigma-Aldrich), non-capacitating control. CAP: modified version of Brackett and Oliphant, 1975 extender. TALP: modified version of Tyrode’s extender. TYR: a classical Tyrode’s formulation. Different superscript letters (a, b, c) within the same row indicate significant differences among treatments within each specific incubation time (*p* < 0.05). Superscript numbers indicate significant differences between time points within the same treatment (*p* < 0.05). Superscript letters and numbers are separated by a semicolon (e.g., a;1–3), where hyphens represent ranges of time points sharing non-significant differences. Data are shown as mean ± standard errors of the mean (SEM; *n* = 8 pools).

## Data Availability

The data presented in this study are available on request from the corresponding author. The data are not publicly available because they form part of an ongoing research project and may be used in future publications.
